# Detection of Highly Divergent Tandem Repeats in the Rice Genome

**DOI:** 10.3390/genes12040473

**Published:** 2021-03-25

**Authors:** Eugene V. Korotkov, Anastasiya M. Kamionskya, Maria A. Korotkova

**Affiliations:** 1 Institute of Bioengineering, Research Center of Biotechnology of the Russian Academy of Sciences, Bld.2, 33 Leninsky Ave., 119071 Moscow, Russia; rifampicin@yandex.ru; 2 MEPhI (Moscow Engineering Physics Institute), National Research Nuclear University, 31 Kashirskoye Shosse, 115409 Moscow, Russia; bioinf@rambler.ru

**Keywords:** tandem repeats, dynamic programming, rice genome

## Abstract

Currently, there is a lack of bioinformatics approaches to identify highly divergent tandem repeats (TRs) in eukaryotic genomes. Here, we developed a new mathematical method to search for TRs, which uses a novel algorithm for constructing multiple alignments based on the generation of random position weight matrices (RPWMs), and applied it to detect TRs of 2 to 50 nucleotides long in the rice genome. The RPWM method could find highly divergent TRs in the presence of insertions or deletions. Comparison of the RPWM algorithm with the other methods of TR identification showed that RPWM could detect TRs in which the average number of base substitutions per nucleotide (x) was between 1.5 and 3.2, whereas T-REKS and TRF methods could not detect divergent TRs with x > 1.5. Applied to the search of TRs in the rice genome, the RPWM method revealed that TRs occupied 5% of the genome and that most of them were 2 and 3 bases long. Using RPWM, we also revealed the correlation of TRs with dispersed repeats and transposons, suggesting that some transposons originated from TRs. Thus, the novel RPWM algorithm is an effective tool to search for highly divergent TRs in the genomes.

## 1. Introduction

The rapid development of sequencing techniques in recent years has allowed determination of complete genome sequences for many eukaryote species [1]. As a result, a large amount of data on various types of nucleotide sequences has been accumulated, leading to challenges in the determination of their functional significance and evolutionary origin. Currently, many computer methods have been developed for the functional annotation of various DNA sequences, including algorithms for the search of coding regions, promoters, transposons, and short and long interspersed nuclear elements (SINE and LINE, respectively) [2]. The identification of satellite DNA tandem repeats (TRs), including minisatellites and microsatellites, is also a part of the genome annotation task. Mini- and microsatellites are short repeats of 6–100 and 2–5 bases long, respectively [3,4]. Between the two, microsatellites are the most prevalent and, consequently, the most studied; they are used as molecular markers, in particular, to assess the genetic diversity of agricultural plant and animal species [5].

The various mathematical methods used to find TRs can be classified into two types [6]. The first one comprises spectral methods, including Fourier transform [7,8,9], Wavelet analysis [10], and information decomposition [11]. These methods make it possible to find TRs with both perfect periodicity (i.e., identical individual repeats) and those containing a large number of DNA base substitutions. However, these methods have a significant limitation as they are very sensitive to nucleotide deletions and insertions (indels), in the presence of which they largely lose the efficiency in detecting TRs because of periodicity phase shifts occurring at the place of the indel less than the repeat length [12]. As a result, spectral methods consider nucleotides before and after the indel in different phases, which significantly limits their performance [13]; consequently, many TRs in eukaryotic genomes can be missed.

The methods and algorithms of the second class are based on dynamic programming, which can efficiently detect indels; therefore, these approaches are more widely used to search for TRs. The dynamic programming-based algorithms such as Tandem Repeat Finder (TRF) [14], Mreps [15], TRStalker [16], ATRHunter [17], T-REKS [18], IMEX [19], CRISPRs [20], and SWAN [21] often employ alignment between all possible pairs of repeats, which, however, imposes certain restrictions on their ability to search for TRs. The problem is that pairwise alignment is limited by the number of the accumulated substitutions per unit (nucleotide or amino acid) designated as *x*. Thus, for amino acid sequences *x* is limited to 2.5 residues [22]. It means that if two compared proteins have a common ancestor but have accumulated over 2.5 substitutions per residue (*x* > 2.5), the dynamic programming methods would not detect statistically significant similarity between them, usually giving the result of <25%. It is believed that the alignment of such sequences is in the twilight zone; therefore, it is not possible to draw a conclusion about their relatedness. A similar situation exists for nucleotide sequences [23], for which the twilight zone is in the range of 38–50% similarity, indicating that *x* in most cases would be less than 1.5. Because of this limitation, mathematical methods and algorithms currently used to search for TRs would identify only those repeats that have accumulated few nucleotide substitutions; as a result, a significant number of TRs present in eukaryotic sequences would be missed.

It is reasonable to assume that the above-mentioned problem can be addressed by using multiple instead of paired alignment of repeats. In fact, the search for TRs in a nucleotide sequence is based on performing multiple alignments, in this case—between all repeats, which makes it possible to find highly diverged TRs, i.e., those with *x* > 1.5. Currently, many mathematical methods such as CLUSTAL [24,25], MAFFT [26,27], and T-COFFEE [28,29] are available for performing multiple alignment. However, most of them still operate by conducting pairwise comparison of the multiple sequences in question through so-called progressive algorithms: They first calculate a statistically significant guide tree and then use it to build multiple alignment. Consequently, such algorithms do not fundamentally solve the problem of searching for highly divergent TRs in eukaryotic sequences. This drawback could be fixed by using multidimensional dynamic programming [30]; however, as this is a so-called Non-deterministic Polynomial (NP)-complete problem [31], its direct solution would require a very long computation time even for only tens of nucleotide sequences.

In our previous studies, we have developed a new mathematical method to search for TRs, which uses a new algorithm for constructing multiple alignments based on the generation of random position weight matrices (RPWMs) [32,33,34]. The RPWM algorithm does not calculate multiple alignment for TRs but utilizes random multiple alignments patterns to find the one that best fits the TRs under study [35]. To search for TRs of length *n* in sequence *S*, we use a PWM containing *n* columns and 4 rows; the PWM is then optimized by applying a genetic algorithm, for which we take the maximum of the similarity function (*F_max_*) for local alignment between sequence *S* and PWM as an objective function [32]. For each length *n*, we determine the best PWM that has the largest *F_max_* value designated as *F_max_*(*n*), i.e., we believe that sequence *S* contains TRs of length *n* for which *F_max_*(*n*) is observed.

In this study, we applied the developed method to search for TRs in the complete genome of rice (*Oryza sativa)* selected as one of the most studied plant genomes, in which the positions of many dispersed repeats and transposons are known [36]. Here, we accomplished three tasks. First, we identified all TRs in the rice genome. Second, we determined correlations of the found TRs with the known genes, dispersed repeats, and transposons. Finally, we compared our method with T-REKS [18] and TRF [14] for the efficiency of TR search. The results indicated that the RPWM algorithm could identify highly divergent TRs (*x* > 1.5), whereas T-REKS and TRF methods were unable to detect TRs with *x* > 1.5. By using our method, we revealed that 5% of the rice genome contained TRs, most of which had a length of 2–3 bases.

## 2. Materials and Methods

### 2.1. RPWM Algorithm

We searched for TRs in chromosome sequences from the rice genome (ftp://ftp.ensemblgenomes.org/pub/plants/release-46/fasta/oryza_sativa/DNA/, (accessed on 18 November 2019); each chromosome was analyzed separately. The algorithm used for TR search is described in detail in [37]. The main steps of the algorithm are outlined below.

Step 1. In each chromosome, we used a window of length *L* = 650 nucleotides (nt) denoted as sequence *S*; nucleotides a, t, c, and g in this sequence were replaced by numbers 1, 2, 3, and 4, respectively. The window moved along the chromosome with a step of 10 bases. Coordinate *k* marked the beginning of sequence *S* in the analyzed chromosome. To search for TRs of length *n* (*n* = 2–50) in sequence *S*, we used an iterative procedure. First, we introduced vector *V* which had *N* elements and made all its values equal to 0; the *mF_max_* value was also 0, the repeat length *n* was 2, and *k* was 1.

Step 2. Next, we created set *Q* of RPWMs with volume *N* = 500. To obtain RPWMs, we used a random number generator to construct sequence *S*_1_ of length *L*_1_ = 10,000 nt with an equally probable nucleotide composition. Then, we constructed sequence *S*_2_ of the same length as *S*_1_, which contained a region {1, 2, 3, …, *n*} of length *n* repeated *L*_1_/*n* times, and filled in the frequency matrix as *M*(*s*_1_(*i*),*s*_2_(*i*)) = *M*(*s*_1_(*i*),*s*_2_(*i*))+1 for *i* = 1, 2, …, *L*_1_. Based on matrix *M*, we calculated matrix *M*_1_ as:

(1) $$m_{1}(i,j)=\frac{m(i,j)-L_{1}p(i,j)}{\sqrt{L_{1}p(i,j)(1-p(i,j))}}$$

where *L*_1_*p*(*i*,*j*) is the expected number of base *i* in position *j*, *m*_1_(*i*,*j*) is the cell of matrix *M*_1_ from set *Q*, *p*(*i*,*j*) =$p\left(i,j\right)=x(i)y(j)/L_{1}^{2}$, $x(i)=\sum_{j=1}^{n}m(i,j)$, and $y(j)=\sum_{i=1}^{4}m(i,j)$. 

Step 3. All matrices from set Q were transformed so that $R^{2}=\sum_{i=1}^{4}\sum_{j=1}^{n}m_{1}{\left(i,j\right)}^{2}$ and $K_{d}=\sum_{i=1}^{4}\sum_{j=1}^{n}m_{1}(i,j)p_{1}(i)p_{2}(j)$ were equal for all matrices. Here, *p*_1_(*i*) = *p*(*l*)*p*(*m*), (*l*,*m* ∈ {a,t,c,g}), *p*(*l*) and *p*(*m*) are the probabilities of *l* or *m* type nucleotides in sequence *S*, and $p_{2}\left(j\right)=1/n$. *R*^2^ and *K_d_* were the same and equal to 55,000/(*n*^0.61^) and −1.5, respectively; the power of 0.61 was chosen experimentally. The matrix transformation algorithm is described in detail in [32]. 

Step 4. At this step, we excluded areas with triplet periodicity from consideration. To do this, we compared sequence *S* with sequence *S*_3_, which contained numbers 123 in tandem and had the same length as sequence *S*. After that, we filled in matrix *M*3 with dimension (3,4) as *M*3(*s*_3_(*i*), *s*(*i*)) = *M*3(*s*_3_(*i*), *s*(*i*))+1 for all *i* from 1 to 650, where *s*_3_(*i*) and *s*(*i*) are elements of sequences *S*_3_ and *S*, respectively, and calculated

(2) $$2I=2\{\sum_{i=1,}^{3}\sum_{j=1}^{4}m_{3}(i,j)-\sum_{i=1}^{3}x(i)-\sum_{j=1}^{4}y(j)+L\ln L\}$$

where $x(i)=\sum_{j=1}^{4}m_{3}(i,j)$, $y(j)=\sum_{i=1}^{3}m_{3}(i,j)$, and $m_{3}(i,j)$ is an element of matrix *M*3. 2*I* can be viewed as a random variable; it has χ2 distribution with 6 degrees of freedom [38]. We calculated the argument of normal distribution $x_{3}=\sqrt{4I}-\sqrt{11.0}$, which reflects the level of triplet periodicity in sequence *S* without introducing indels [39]; only sequences with $x_{3}$ < 3.0 were considered, which removed all chromosomal regions with a clear triplet periodicity from analysis, including most coding regions. If $x_{3}$ ≥ 3.0, then we increased *k* by 10 and returned to step 1.

Step 5. Next, we applied the genetic algorithm for the matrices from set *Q* to create matrix *Q_max_*, which had the greatest similarity function *F_max_* with sequence *S*. The procedure is described in detail in [32]. For the genetic algorithm, the matrices were organisms and *F_max_*, which was determined for each matrix from set *Q*, was used as an objective function:

(3) $$\begin{array}{l} F(i,j)=\max\left\{\begin{array}{l} F(i-1,j-1)+q(s(i),s_{2}(j))\\ F_{x}(i-1,j-1)+q(s(i),s_{2}(j))\\ F_{y}(i-1,j-1)+q(s(i),s_{2}(j)) \end{array}\right\} \end{array}$$

(4) $$\begin{array}{l} F_{x}(i,j)=\max\left\{\begin{array}{l} F(i-1,j)-d\\ F_{x}(i-1,j)-e \end{array}\right\} \end{array}$$

(5) $$\begin{array}{l} F_{y}(i,j)=\max\left\{\begin{array}{l} F(i,j-1)-d\\ F_{y}(i,j-1)-e \end{array}\right\} \end{array}$$

Here, *s*(*i*) and *s*_2_(*i*) are elements of sequences *S* and *S*_2_, respectively, *i* = 1, 2, …, *L* and *j* = 1, 2, …, *L* are elements of matrix *Q_max_*, *d* is the cost of creating an indel, and *e* is the cost of continuing the indel; *d* = 25.0 and *e* = 6.0. 

To fill in matrix *F*(*L*,*L*), we defined the initial conditions as *F*(0,*i*) = *F*(*i*,0) = 0 and searched for the greatest *F_max_* on the boundaries of matrix *F*(*L*,*i*) and *F*(*i*,*L*) (*i* = 1, …, *L*). *F_max_* determined for each matrix from set *Q* was inserted into vector *V*, which contained *N* elements (the number of matrices in set *Q*) and which was then ranked in the descending order of the elements; as a result, the maximum and minimum elements were *V*(1) and *V*(*N*), respectively.

Step 6. At this step, we introduced mutations into the matrices from *Q* set. For this, in a randomly chosen matrix we randomly chose cell *m*(*i*, *j*) (with 1 ≤ *i* ≤ *n*, 1 ≤ *j* ≤ 4) and then replaced the cell with a random number in the range from -10 to +10; this procedure was used to introduce random mutations in 5% of the matrices. In addition, we created a descendant matrix through a process described in detail in [32]). Briefly, the matrix with the minimum value *V*(*N*) was excluded from set *Q* and two matrices were randomly selected from set *Q* and considered as parents; the probability of a matrix to be chosen as a parent increased linearly from the minimum to the maximum element of vector *V*. From the parts of the two parent matrices, we created a new matrix considered as a descendant, which was inserted into set *Q*.

Step 7. Then, we checked that *V*(1) ≥ *mF_max_*; if this was true, then *mF_max_* = *V*(1), the matrix corresponding to *V*(1) was designated as *mQ*, and we moved to step 2. In case when *V*(1) < *mF_max_* was observed up to four consecutive times, we went to step 2 without changing *mF_max_* and *mQ*; if it was observed five consecutive times, the cycle was interrupted. Usually, 10^4^ such cycles were enough for the algorithm to stop; if it did not happen, then we stopped it manually in order to reduce the computation time, as there were cases when the cyclic process was performed 10^5^ times.

Step 8. Next, we aligned sequence *S* with the found matrix *mQ* using dynamic programming. We filled in the matrix for function *F* (Formulas (2)–(4)) as well as the so-called matrix of inverse transitions, *Fb;* in each cell of matrix *Fb*(*i*,*j*), we wrote the coordinates of that cell of matrix *F* from which we got into cell F(*i*,*j*). In *Fb*(*i*,*j*), we found a point with coordinates (*i_m_*, *j_m_*), where *F*(*i_m_*,*j_m_*) = *mF_max_*, then moved from point (*i_m_*,*j_m_*) to point (*i*_0_,*j*_0_), where *F*(*i*_0_,*j*_0_) = 0, and performed alignment between sequences *S* and *S*_2_. Thus, for the repeat of length *n*, we obtained matrix *mQ*, *mF_max_*, coordinates *i*_0_ and *i_m_*, and the alignment between sequences *S* and *S*_2_.

Step 9. In this step, we increased *k* by 10 bases and went to step 5; this was done up to *k* = *L*-649. As a result, we obtained function *mF_max_*(*k*) for the repeat of length *n*, which was designated as *mF_max_*(*k*,*n*). Next, we searched for the local maxima of *mF_max_*(*k*,*n*), i.e., selected such values of *k* at which *mF_max_*(*k*,*n*) > *mF_max_*(*k + i*,n) for any *i* = −64, −63, …, −1 and any *i* = 1, …, 63, 64; then, all such *mF_max_*(*k + i*,n) were equated to zero. 

Step 10. We increased *n* by one and went to step 2 until *n* ≤ 50; as a result, we obtained a set of local maxima *mF_max_*(*k*,*n*) for repeat *n*. Then, we chose local maxima for *mF_max_*(*k*,*n*); these were *k* and *n* for which *mF_max_*(*k*,*n*) > *F*_0,_ as well as all *mF_max_*(*k+i*,*n+j*) for any *i* = −64, −63, …, −1 and *i* = 1, …, 63, 64, and any *j* ≤ 50 and *j* ≠ *n*. All found local maxima (their number denoted as *T*) have the length of repeat *n*, matrix *mQ*, *mF_max_*, coordinates *i*_0_ and *i*_m_, and the alignment between sequences *S* and *S*_2_ for each local maximum.

### 2.2. Choice of the F0 Threshold Value 

The total number of the local maxima (*T*) represents the sum of the local maxima which owe their origin to random factors (*T*rand) or to the periodicity of nucleotide sequences (*T*real). To choose the F0 threshold, we randomly shuffled chromosome sequences from the rice genome, determined *T*rand, and established F0 so that the false discovery rate (FDR) = *T*rand /(*T*rand+*T*real) was <0.01; at this condition, F0 = 390.

### 2.3. Evaluation of the Statistical Significance of Periodicity for Model Sequences

To estimate the statistical significance of the periodicity of the repeat with length *n*, we randomly shuffled sequence *S* and followed steps 1–5 (Section 2.1) to obtain *mF_max_* for the random sequence *S* by aligning it with sequence *S*_2_, which had the form {1, 2, 3, …, *n*}*L*/*n*. Then, we shuffled sequence *S* again and re-calculated *mF_max_*; this procedure was performed 200 times. As a result, we obtained a set of 200 *mF_max_* values, for which we calculated mean $\bar{mF_{max}}$, variance *D*(*mF_max_*), and statistical significance *Z*(*n*): *Z*(*n*) = (*mF_max_* − $\bar{mF_{max}}$)/$\sqrt{D(mF_{max})}$. This calculation was performed for sequences containing artificially created repeats with *n* from 2 to 50 nt. 

## 3. Results

### 3.1. Analysis of DNA Sequences with Artificial Periodicity

**Figure 1:**
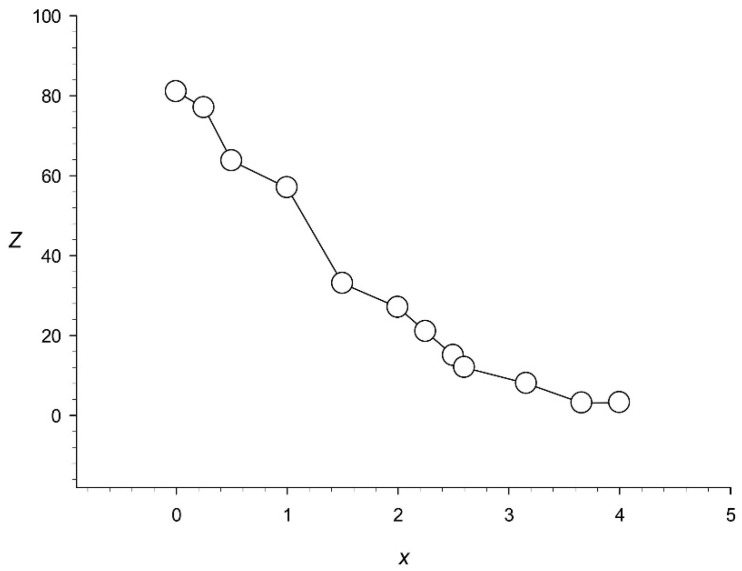
Dependence of statistical significance *Z* on the average number of substitutions per nucleotide *x* between any two repeats. A total of 50 insertions and 50 deletions of 1 nt were made in an artificial sequence of 3000 nt, which contained 100 repeats of 30 nt each.

To determine the number of random mutations at which tandem periodicity can be detected, we first examined an artificially created sequence of 3000 nt long, which contained 100 TRs of 30 nt each. Then, 50 insertions and 50 deletions of 1 nt were made in the analyzed sequence. For this, we randomly selected a nucleotide (from 1 to 3000), which was then substituted with any of the four nucleotides (A, T, C, or G) with the probability of 0.25. As a result, we created 12 sequences carrying from 0 to 6000 substitutions (Figure 1). For each such sequence designated as *Ts*(*l*) (*l* = 1, …, 12), we also defined vector $y^{l}(k)$, *l* = 1, 2, …, 3000, which showed the number of base changes made at position *k* of sequence *Ts*(*l*), and calculated the average number of base substitutions *x* existing between any two repeats in the analyzed sequence:

(6) $$x=\frac{\sum_{i=0}^{99}\sum_{j=0}^{99}\{\sum_{k=1+30i}^{30(i+1)}y(k)+\sum_{k=1+30j}^{30(j+1)}y(k)\}}{30L}.$$

Here, *L* is the total number of unique pairs of different TRs and 30 is the length of the TR; in this case, $L=\sum_{i=1}^{99}i=9900$. Coordinates (*i* + 1) and (*j* + 1) indicate the beginning of TRs in sequence *Ts*(*l*). 

Figure 1 shows that the threshold *Z* = 8.0 is reached at *x* = 3.2, indicating that the method we developed could find highly divergent repeats in the genome.

### 3.2. Comparison of TRF, T-REKS, and RPWM Programs

**Figure 2:**
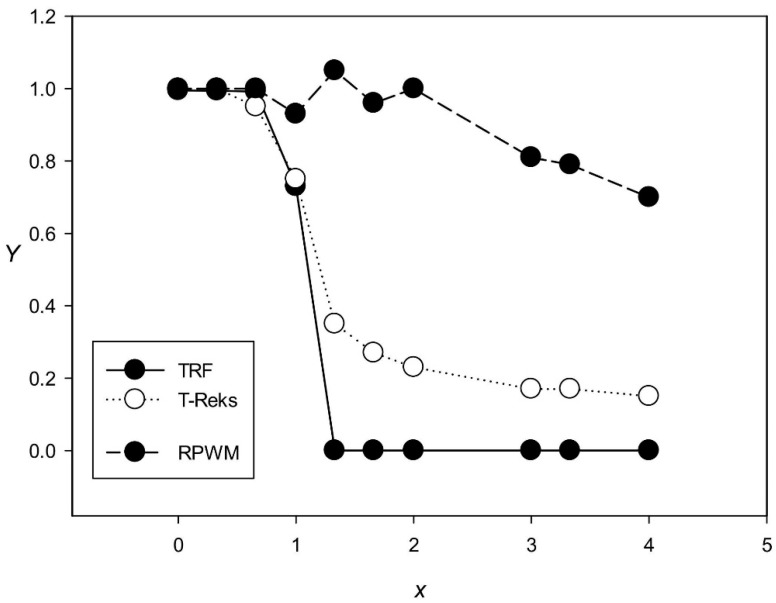
Dependence of the proportion of identified tandem repeats (*Y*) on the number of introduced base substitutions (*x*) for the Tandem Repeat Finder (TRF), T-Reks, and random position weight matrices (RPWMs) programs. The analyzed sequences contained 100 repeats 6 nt long; each sequence had a different number of base substitutions along with 5 insertions and 5 deletions at random positions.

Next, we compared the performance of the RPWM method with that of TRF [14] and T-REKS [18] in regard to TR search. For this, we generated test sequences containing 100 TRs with the length of 6 nt. Each created sequence had a different number of base substitutions, for which we calculated *x* using formula (6), along with 5 insertions and 5 deletions at random positions. We also added random sequences of 300 nt long at the beginning and the end of the generated sequence; as a result, each generated sequence contained 1,200 nt. Thus, a set of sequences for 0.0 < *x* < 4.0 was obtained and used to compare the performance of the TRF, T-REKS, and RPWM programs in determining the number (*K*) of 6 nt repeats for different *x*. Figure 2 shows that that the proportion of repeats (*Y*(*x*) = *K*/100) detected by TRF decreased sharply for *x* > 1.0, reaching 0 for *x* > 1.3. A similar decrease was observed for T-REKS, when *Y*(*x*) fell below 0.4 for *x* > 1.3 and was 0.2 for *x* > 2.0. At the same time, RPWM could detect significantly more TRs than TRF and T-REKS, as evidenced by *Y* = 0.8 for x = 3.0 and *Y*(*x*) = 0.65 for *x* = 4.0 (Figure 2). These results indicate that RPWM is an efficient algorithm for identifying TRs with a significant number of substitutions (*x* > 1.5).

It should also be noted that in the test sequences, TRF and T-REKS detected TRs with lengths other than 6 nt. In such cases, the results were considered incorrect and were not included in *K* and, consequently, in Figure 2.

We next compared the efficiency of searching for TRs in the first chromosome of the rice genome. To do this, we selected repeats of different lengths from the genome and analyzed their presence in the first chromosome; as a result, 8276 regions containing repeats of 2 to 50 nt were detected by the RPWM method. Then, we randomly shuffled he first chromosome and repeated the search, which detected 18 regions with periodicity, indicating that FDR = *T*rand/(*T*rand + *T*real) for RPWM was 18/8276 ≈ 0.25%. 

The 8276 regions detected by RPWM in the first rice chromosome were then analyzed by TRF and T-REKS. We considered that a region was found with periodicity if the length of the period coincided and the intersection of these regions was at least 50%. Out of the 8,276 regions, TRF identified 266 that had the same repeat length as found by RPWM, whereas after random shuffling, it detected 16 regions with TRs, which gave the FDR of 16/266 ~ 6%, indicating that even with a higher FDR, TRF could find no more than 3% of TR-containing regions detected by RPWM. T-REKS found 1,276 regions for *p_sim_* = 0.77 and 28 regions after random shuffling, which gave the FDR = 28/1276 ~ 2% (*p_sim_* is similarity coefficient which is calculated by T-REKS). At smaller *p_sim_* values, we found a very large number of regions for random sequences; thus, at *p_sim_* = 0.70, the number was 897, which indicated that the reduction of *p_sim_* even worsened the result.

### 3.3. Search for TRs in the Rice Genome

We applied the RPWM algorithm to analyze all 12 rice chromosomes. The numbers of regions with periodicity in each chromosome are shown in Table 1. The total number of such regions in the genome was 76,174 and their total length was slightly over 19 million bases, which is more than 5% of the total rice genome. 

**Figure 3:**
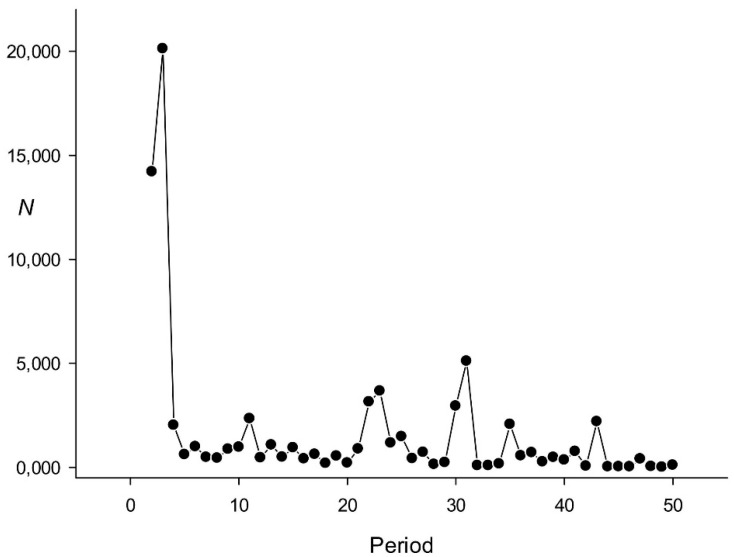
Distribution of regions with TRs in the rice genome according to the repeat length.

The distribution of repeats with a certain length in the rice genome is shown in Figure 3. The results indicated that most repeats had a length of 3 nt. Step 4 of the algorithm excluded all DNA regions with triplet periodicity without indels. The presence of TR with *n* = 3 in Figure 2 indicated that there were a large number of regions where only triplets with indels could be detected, which is confirmed by the fact that many potential reading frame shifts that have been identified are associated with the presence of indels in the triplets [40]. Figure 3 also shows that a significant number of repeats were 2 nt long; other peaks were detected at 11, 22–23, and 31 nt. These data are consistent with the pitch of the DNA helix in the B form.

**Figure 4:**
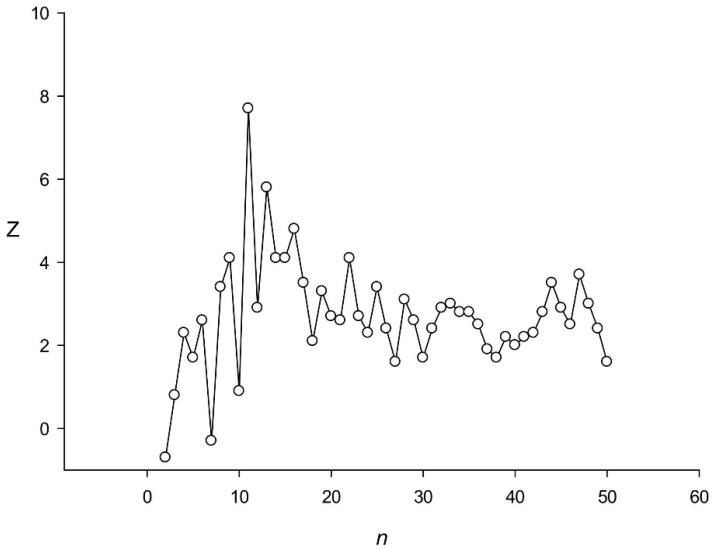
Dependence of *Z*(*n*) for the DNA region from 2,630,061 to 2,630,589 nt of the first rice chromosome. *mF_max_*(11) = 610.3.

Figure 4 shows the distribution of the *Z* value for repeats found in the rice genome. It can be seen that *Z*(11) = 7.9 was the maximum for repeats from 2 to 50 nt. Table 2 displaying the resulting alignment for sequence *S* indicates that it was very difficult to detect periodicity without indels. Table 3 shows the *mQ*(11) matrix for the found periodicity; each column of the matrix has one or more positive values corresponding to nucleotides, the number of which in a given position of the repeat was greater than expected for a random sequence. The consensus sequence for the repeat of 11 nt was taggagtggca, if constructed from the positive values of the matrix. 

Next, we examined the correlation of TRs with exons on rice chromosomes. The regions were considered as intersecting if they overlapped by at least 80%. The results for different chromosomes are shown in Table 4 (third column). Then, we randomly shuffled TR locations in the chromosomes and again analyzed their intersections with exons (Table 4, fourth column). The results revealed that the number of overlaps between exons and randomly located TRs exceeded that of overlaps between exons and actual TRs in most chromosomes, indicating a tendency for TRs (for *n* ≠ 3) to be located outside the coding regions.

We hypothesized that the found repeats could be associated with LTRs, mobile elements, and SINE and LINE. Therefore, we analyzed the intersection of TRs with transposable elements (TEs) identified in a previous study [36] using the same criterion of intersection (≥ 80%). We randomly shuffled TE locations on each rice chromosome and counted the number of overlaps between TRs and TEs for each shuffle to calculate $X=\{C-\bar{C}\}/\sqrt{D(C)}$, where C is the number of intersections of TRs with TEs in the rice genome, $\bar{C}$ is the expected number of intersections, and *D*(*C*) is the variance for the expected number of intersections (Table 5). The larger X, the more the found number of intersections of TRs with TEs differs from the number of intersections that is expected for a random (independent) arrangement of TRs and TEs in the rice genome. The results indicated that the location of some TEs was strongly correlated with that of TRs, which was especially evident for Centro/tandem, DNAnona/CACTA, DNAnona/MULE and DNAauto/CACTG as well as some other transposons. This means that a large number of TRs are present in the TEs sequences.

We have created a database for the detected TRs (http://victoria.biengi.ac.ru/cgi-bin/indelper/index.cgi/, (accessed on 18 October 2020), where the user can search for repeats by the length, location in a specific rice chromosome, and level of statistical significance.

## 4. Discussion

This work shows that about 5% of the rice genome contains TRs, including highly divergent ones that can be identified only by the method developed in this study. According to our calculations (Section 3.2), no more than 15% of the found TRs could be detected by previously developed methods.

At the same time, a small number of TRs for *n* < 10 are better detected by T-REKS or TRF. The reason for this is that we are using dynamic programming to find alignment (Formulas (3)–(5)). Only TRs with a total length of more than 20 nucleotides can be effectively detected using this procedure. This means that the number of TRs that can be detected by the RPWM is approximately 20/*n*, where *n* is the length of the period. For *n* ≥ 10 nucleotides, RPWM effectively finds all periods.

The example shown in Figure 4 and Table 2 contains highly diverged TRs. Consensus sequence has an average similarity of 57% with repeats. In addition to base substitutions, there are indels here. Two hypotheses can be assumed. First, there were perfect TRs that accumulated over time many base replacements and also indels. Many authors have previously noted that TRs mutate very rapidly [41]. Second, TRs can bind to different proteins. Because of this, we see TRs 11 bases in length, which indicates the possibility of binding to some proteins with zinc fingers [42]. These two hypotheses may explain the origin of the significant number of highly divergent TRs found in this work.

The repeat with a length of 3 nt was the one most frequently identified. Although triplets without indels were not considered (Section 2.1, step 4), still more than 20 thousand regions with triplet repeats containing indels were observed (Figure 3). It was also possible to calculate the total number of regions with triplet periodicity within the coding sequences of the rice genome, which was 14,534 (Table 4), indicating that 75% of the detected regions containing triplet repeats with indels are in the coding sequences, where they may represent potential frame shifts [40]. Therefore, it would be more correct to say that most TRs we identified had a repeat length of 2 nt. A small peak for repeats of 11 nt (Figure 3) suggests that some of the detected sequences can interact with proteins [43]; the same can be also expected for TRs of 20 and 30 nt long.

Many of the found sequences (54,434 out of 76,174) belong to transposons or previously known repeats (Table 5), which may indicate an organic relationship between TRs and transposonomy as suggested by Paço et al. [44], who also discussed a possibility of TR conversion into genes. The latter hypothesis is supported by our results, which revealed that many regions with triplet periodicity were located within the genes.

Significantly divergent TRs present in the rice genome can be highly polymorphic and, therefore, may be used as molecular markers for genetic analysis [45], for example, in plant breeding [46] or in the investigation of the evolutionary divergence among species [47], as well as in other areas.

## 5. Conclusions

Here, we used the novel RPWM algorithm to search for TRs in the rice genome. The developed method makes it possible to find TRs with an average number of substitutions between any two repeats (*x*) up to 3.2. In comparison, the previously used methods TRF and T-REKS could correctly identify TRs with *x* ≤ 1.5 and were able to find only about 15% of TRs detected in the rice genome by the RPWM algorithm. A total of 76,174 sequences with repeats of 2–50 nt found in rice chromosomes occupy about 5% of the rice genome; most of these TRs are 2 and 3 nt long and 54,434 of them are present in already known repeats and transposons.

## Figures and Tables

**Table 1:** The numbers of regions with TRs of different lengths detected in rice chromosomes.

1	2	3	4	5	6	7	8	9	10	11	12
8277	6960	6679	6999	6073	6559	6234	6083	4925	4886	6397	6102

**Table 2:** Alignment of sequences *S*2 and *S* for the DNA region from 2,630,061 to 2,630,589 nt of the first rice chromosome. Numbers 10 and 11 in sequence *S*_2_ are replaced with a and b.

NO	1234567...8....9a.b
1	CATTTGA...A....TC.G.
2	CAGGATT...G....AA.A.
3	AAACGGA...G....GA.A.
4	TAGGAAA...A....AC.A.
5	CAGGAAT...C....TG.A.
6	TAGGAAT...G....CA.A.
7	GTGTAAA........AC.A.
8	GAGGATT...GCAAAAC.A.
9	CAGGAAA...A....AC.A.
10	TAGGAAT...G....AC.C.
11	GTTTAATTGGA....CC.A.
12	CAGGAAA...A....AC.A.
13	CAGGAAT...C....AG.A.
14	TGAGAGA...G....AT.A.
15	GACTTAG...G....GC.C.
16	CCTTTGA...A....TC.A.
17	TAGGAAT...G....AA.A.
18	AAACGGA...G....GA.A.
19	TAGGAAA...A....AC.A.
20	TATGATT...A....TG.A.
21	CAGGAAT...G....TA.A.
22	GTGTAAA........AC.A.
23	GAGGATT...GCAAAAC.A.
24	CAGGAAA...A....AC.A.
25	TAGGAAT...G....AC.CG
26	TTTGATT...GGA..CC.A.
27	TAGGAAA...A....AC.A.
28	CAGGAAT...T....TG.A.
29	GGAGAGA...T....AA.A.
30	GACTCAA...A....GG.A.
31	TTTCTTC...C....AT.G.
32	AGGTTCT...A....CC.T.
33	CATGTTA...A....AA.T.
34	TCCTCCA...A....AA.C.
35	TTGTATG...G....GA.A.
36	GAGGCAT...T....CC.A.
37	TAGGAAT...T....TC.A.
38	TAAGATT...C....AATA.
39	GGGTTCA...T....TC.A.
40	TTTGATT...C....AA.A.
41	GGGCTTT...G....TA.G.
42	GAAAAAT...T....CCTA.
43	TAGGAAT...A....AA.A.
44	T

**Table 3:** Matrix *mQ*(11) for the TRs in the region from 2,630,589 to 2,630,061 nt of the first rice chromosome.

	1	2	3	4	5	6	7	8	9	10	11
A	1.4	−4.8	−4.0	−0.7	−5.3	6.0	−9.4	1.9	−7.3	−9.4	3.6
T	−1.3	8.1	−2.5	−2.5	−6.3	−7.4	5.3	−1.9	−1.3	−3.5	−4.1
C	5.0	−4.3	−0.8	0.6	12.4	−0.8	0.6	−3.6	−3.6	−2.2	−4.3
G	4.4	−6.7	1.2	−3.9	−3.9	−6.7	−0.5	−5.0	7.4	11.3	−5.0

**Table 4:** Intersection between exons and regions containing tandem periodicity in rice chromosomes.

Chromosome	Number of Exons	Number of Overlaps	Average Number of Overlaps at Random Locations
1	28,791	854	2297
2	23,456	1665	1876
3	25,583	1819	1956
4	17,446	1458	1462
5	16,256	1259	1295
6	16,038	1316	1329
7	14,744	1167	1180
8	12,888	1016	1046
9	10,563	964	822
10	9992	910	815
11	11,092	1142	932
12	10,936	964	891

**Table 5:** Intersection of regions with periodicity and known transposons and repeats. *X* is the normal distribution argument of the identified intersections calculated using the Monte Carlo method.

Repeat Name	Number of Overlapping Repeats	Number of Expected Overlapping Repeats	*X*
DNAnona/Helitron	4721	2222	53.0144
DNAnona/unknown	99	244	−9.2827
MITE/Tourist	1567	2346	−16.0832
MITE/Stow	1258	2112	−18.5828
DNAauto/MULE	1662	962	22.5689
DNAnona/MULE	8850	2648	120.5238
LINE/unknown	2520	1218	37.3067
LTR/Gypsy	14,664	9534	52.5388
DNAnona/hAT	2273	1008	39.8438
DNAnona/MULEtir	468	486	−0.8165
DNAnona/Tourist	292	144	12.3333
LTR/Copia	2830	1692	27.6657
DNAauto/CACTA	2141	688	55.3951
SINE/unknown	440	404	1.7911
DNAnona/CACTA	3144	688	93.6341
DNAauto/hAT	315	146	13.9865
DNAnona/PILE	231	120	10.1329
DNAauto/PILE	99	108	−0.8660
LTR/TRIM	339	172	12.7336
DNAauto/Helitron	81	118	−3.4061
Evirus/ERTBV-C	5	4	0.5000
LTR/unknown	175	50	17.6777
DNAnona/CACTG	1189	234	62.4303
DNAauto/CACTG	3367	958	77.8313
LTR/Solo	17	6	4.4907
DNAauto/MLE	68	72	−0.4714
Evirus/ERTBV-B	9	30	−3.8341
Evirus/ERTBV-A	11	28	−3.2127
Evirus/ERTBV	3	12	−2.5981
DNAauto/POLE	68	52	2.2188
DNAnona/POLE	81	64	2.1250
DNAnona/MLE	12	8	1.4142
Centro/tandem	1351	80	142.1021
Satellite/rice	84	26	11.3747

## Data Availability

http://victoria.biengi.ac.ru/cgi-bin/indelper/index.cgi (accessed on 22 March 2021)
